# Anti-oxidant, anti-apoptotic, and protective effects of myricitrin and its solid lipid nanoparticle on streptozotocin-nicotinamide induced diabetic nephropathy in type 2 diabetic male mice

**DOI:** 10.22038/IJBMS.2023.71189.15462

**Published:** 2023

**Authors:** Reza Mohebbati

**Affiliations:** 1 Department of Physiology, Faculty of Medicine, Gonabad University of Medical Sciences, Gonabad, Iran; 2 Applied Biomedical Research Center, Mashhad University of Medical Scinces, Mashhad, Iran

A current study entitled “Anti-oxidant, anti-apoptotic, and protective effects of myricitrin and its solid lipid nanoparticle on streptozotocin-nicotinamide induced diabetic nephropathy in type 2 diabetic male mice” that was published in this journal by Ahangarpour (2019) states that glomerular filtration rates (GFR) declined in the diabetic nephropathy (DN), while according to many articles it was boosted in a short time.

One of the most important problems with this paper, according to the documentation presented, is that the model of diabetic nephropathy is not well established. This can make the whole work especially the positive effect of drugs on diabetic nephropathy problematic. It is certain that, when the disease model is not well established, the positive effect of a drug cannot be reported as a result. In the first step, it is always very important to ensure the creation of the disease model, so that the result of the work reflects the correct creation of the model. 

Thongrung *et al*. in a laboratory investigation concluded that creatinine clearance (GFR) was increased significantly 28 days after DN induction by streptozotocin (STZ). This work was performed last year (2022) with the same method for DN induction (1). This mechanism seems to be reasonable because DN in a short period (28 days) results in a permanent loss of filtering nephrons, which is compensated by hyperfiltration of those remaining. Also, another study performed by Veelken *et al*. has shown that inulin clearance (GFR) increased in diabetic rats after 2 weeks (3129 ± 309 μl/min versus 2297 ± 264 μl/min in diabetic rats) (*P*<0.05) (2). Luippold *et al*. also performed hyperfiltration in diabetic rats after 14 days (3). In diabetic animals, GFR significantly increased in the early phase (Stage 1) (4). According to studies, this stage can last up to 14 weeks in diabetic animals (5). This increase can have several reasons, resulting in nephropathy. One of the most important reasons is increased proximal glucose reabsorption by sodium glucose transporter-2 (SGLT-2), which impairs the normal tubulo-glomerular feedback mechanism (6). It is important to note that total renal filtration (GFR) is affected by single nephron GFR and by the number of functioning glomeruli. Then, when the number of functioning glomeruli is substantially reduced, the total GFR may be within the normal range despite single glomerular hyperfiltration. Thus, it can be concluded that although the level of creatinine in the plasma sometimes increases in some nephropathies, GFR is high. According to the GFR formula, increased serum creatinine can result in GFR reduction but in some situations, this reduction of GFR is strongly affected by the increase in the urine volume (7).

Another ambiguity of this study is the increased amount of sodium in the urine, while the amount of sodium in the serum has also increased. According to studies conducted in this field, one of the reasons for the increase in glomerular filtration in the first stage of diabetes is the increase in glomerular hydrostatic pressure, which occurs as a result of the activation of the Renin-angiotensin-aldosterone system (RAAS). Therefore, it is logical that the secretion of aldosterone causes an increase in sodium in the blood and a decrease in its excretion (8).

## Conflicts of Interest

None. 
